# Dynamic Random Walk and Dynamic Opposition Learning for Improving Aquila Optimizer: Solving Constrained Engineering Design Problems

**DOI:** 10.3390/biomimetics9040215

**Published:** 2024-04-04

**Authors:** Megha Varshney, Pravesh Kumar, Musrrat Ali, Yonis Gulzar

**Affiliations:** 1 Rajkiya Engineering College (AKTU, Lucknow), Bijnor 246725, India; megha.math21@recb.ac.in (M.V.);; 2 Department of Basic Sciences, Preparatory Year, King Faisal University, Al Ahsa 31982, Saudi Arabia; 3 Department of Management Information Systems, College of Business Administration, King Faisal University, Al Ahsa 31982, Saudi Arabia; ygulzar@kfu.edu.sa

**Keywords:** aquila optimizer, dynamic random walk, dynamic opposite learning, engineering design problems

## Abstract

One of the most important tasks in handling real-world global optimization problems is to achieve a balance between exploration and exploitation in any nature-inspired optimization method. As a result, the search agents of an algorithm constantly strive to investigate the unexplored regions of a search space. Aquila Optimizer (AO) is a recent addition to the field of metaheuristics that finds the solution to an optimization problem using the hunting behavior of Aquila. However, in some cases, AO skips the true solutions and is trapped at sub-optimal solutions. These problems lead to premature convergence (stagnation), which is harmful in determining the global optima. Therefore, to solve the above-mentioned problem, the present study aims to establish comparatively better synergy between exploration and exploitation and to escape from local stagnation in AO. In this direction, firstly, the exploration ability of AO is improved by integrating Dynamic Random Walk (DRW), and, secondly, the balance between exploration and exploitation is maintained through Dynamic Oppositional Learning (DOL). Due to its dynamic search space and low complexity, the DOL-inspired DRW technique is more computationally efficient and has higher exploration potential for convergence to the best optimum. This allows the algorithm to be improved even further and prevents premature convergence. The proposed algorithm is named DAO. A well-known set of CEC2017 and CEC2019 benchmark functions as well as three engineering problems are used for the performance evaluation. The superior ability of the proposed DAO is demonstrated by the examination of the numerical data produced and its comparison with existing metaheuristic algorithms.

## 1. Introduction

Global optimization is a term used to characterize several scientific and engineering problems that can be resolved using different optimization techniques. These days, the preferred methods for global optimization are metaheuristic algorithms (MAs) since they are protected against local maximum efficacy by their stochastic and dynamic nature [1]. Genetic Evolution [2], Differential Evolution (DE) [3], Particle Swarm Optimization (PSO) [4], Reptile Search Algorithm (RSA) [5], Whale Optimization Algorithm (WOA) [6], Brain Storm Optimization (BSO) [7], Teaching–Learning-Based Optimization (TLBO) [8], etc., are several MAs that have emerged over the past 20 years. One of the better algorithms is the AO method, which Abualigah proposed in 2021 [9], because it is simple to build, has consistent performance, and few configurable parameters. Its strong optimization capabilities have helped with a variety of global optimization problems, including feature selection [10], vehicle route planning [11], and machine scheduling [12].

The No Free Lunch (NFL) theorem [13] was a significant advancement in the field of nature-inspired algorithms. It is impossible to develop a single optimization algorithm that solves every optimization problem, according to the NFL theorem. To put it simply, even if optimization method “A” is ideally suited for a particular set of problems, there is always a subset of problems on which it would perform poorly. As a result, the NFL theorem provides the area of nature-inspired algorithms life and enables academics to either suggest new algorithms or enhance already existing ones. In order to improve existing algorithms, an effective approach for doing so is hybridization—combining the best aspects of multiple algorithms to create a hybridized algorithm. The present study aims to combine the benefits of better exploration and the efficiency of maintaining a balance between exploration and exploitation by improving the AO, DOL, and DRW techniques. This is achieved by drawing inspiration from the advantages of improving the algorithm.

The new NIOA, called Aquila Optimizer, uses the Aquila bird’s hunting strategy in an attempt to discover the best solution to an optimization problem. AO is capable of handling a broad range of optimization problems [14]. The first drawback of this algorithm is premature convergence, which happens when the algorithm has a stagnation issue and is unable to explore the whole search space during the process. The second drawback is its low computational efficiency. This provides a poor ideal solution and also prevents the algorithm from searching the whole search space. Aquila Optimizer takes a longer time to converge and to reach the ideal solution than other existing metaheuristic algorithms. Therefore, in the current study, Aquila Optimizer is enhanced so that it can explore the more promising areas that are left in the population’s memory. By combining AO with DRW and DOL, suitable harmony between the exploration and exploitation process is formed. The DOL [15] method with its asymmetric and dynamic search space exhibits a great deal of promise. In the meanwhile, the dynamic opposite number, a random candidate, can be computed quickly and easily. This may enhance the algorithm’s capacity for exploitation and increase the rate of convergence. The DRW [16] approach focuses on iteratively improving a solution by exploring its closer neighborhood because balancing the search for new promising areas with refining solutions within existing areas is the key to metaheuristics. The following are the paper’s contributions:

1. To increase the AO algorithm’s computing effectiveness and capacity for local optimal avoidance, a new DRW technique is put forth.
2. To enhance the algorithm’s performance and balance between exploration and exploitation, the DOL approach is incorporated into AO for the very first time.
3. The performance of DAO is examined on twenty-nine benchmark functions of CEC 2017, ten benchmark functions of CEC 2019, and then on three engineering design problems, and the results are compared with various algorithms.

The following part of the paper is structured as follows: the fundamental ideas of AO, DOL, and DRW are presented in Section 2. The previous work on AO is explained in Section 3. In Section 4, the proposed DAO algorithm is explained. Section 5 presents the experiments and their findings. Section 6 shows the engineering applications. The study’s conclusion is finally presented in Section 7.

## 2. Algorithm Preliminaries

### 2.1. Aquila Optimizer

The Aquila bird’s hunting strategy served as the inspiration for the Aquila Optimizer (AO) metaheuristic optimization technique [9]. AO mimics the four main prey-hunting strategies, explained as follows:

#### 2.1.1. Expanded Exploration

The expanded exploration $x_{1}$ of Aquila Optimizer mimics the high-achieving quickly descending hunting strategy observed in Aquila birds. With this strategy, the bird soars to enormous heights, giving it the opportunity to inspect the whole search area, identify potential prey, and select the ideal hunting place. Equation (1) in [9] provides a mathematical illustration of this strategy.

(1) $$x_{1}^{\left(h+1\right)}=x_{best}^{\left(h\right)}\times \left(1-\frac{h}{H}\right)+\left(x_{M}^{\left(h\right)}-rand\times x_{best}^{\left(h\right)}\right)$$

In Equation (1), the maximum number of iterations is represented as H where h denotes the current iteration. The response for the subsequent iteration indicated as $\left(x_{1}^{\left(h+1\right)}\right)$ is found by the first search in the candidate solution population $\left(x_{1}\right)$. The expression $\left(x_{best}^{\left(h\right)}\right)$ represents the best outcome achieved so far in the $h^{th}$ iteration. A count of iterations is employed through an equation $\left(1-\frac{h}{H}\right)$ to modify the search space’s depth. Additionally, using Equation (2), where N represents the population size and D is the dimension size, the average value of the locations of connected existing solutions at the $h^{th}$ iteration is determined, represented as $x_{M}^{\left(h\right)}$.

(2) $$x_{M}^{\left(h\right)}=\frac{1}{N}\sum_{i=1}^{N}x_{i}\left(h\right),\;\mathrm{for}\;\mathrm{all}\;i=1,2,\ldots ,D$$

#### 2.1.2. Narrowed Exploration

In this approach, the Aquila bird hunts; to track prey, they must fly in a contour-like pattern and execute swift gliding strikes inside a small research region. The primary aim of this methodology $\left(x_{2}^{\left(h+1\right)}\right)$, as expressed mathematically in Equation (3), is to identify a solution for the subsequent iterations.

(3) $$x_{2}^{\left(h+1\right)}=x_{best}^{\left(h\right)}\times Levy\left(D\right)+x_{R}^{\left(h\right)}+\left(v-u\right)\times rand$$

In this approach, Levy(D) is the Levy flying distribution for dimension space D. At the $h^{th}$ iteration, the random solution $\left(x_{R}^{\left(h\right)}\right)$ is taken in the range of $\left[1\;N\right]$, where N is the population size. The Levy flight distribution is calculated using a fixed constant value of s=0.01 and two randomly selected parameters, u and v, which have values between 0 and 1. The mathematical expression for this computation is provided by Equation (4).

(4) $$Levy\left(D\right)=s\times \frac{u\times \sigma }{{\left|v\right|}^{\frac{1}{a}}}$$

Equation (5) finds the value σ, which is obtained using the constant parameter a=1.5.

(5) $$\sigma =\left(\frac{\Gamma \left(1+a\right)\times \sin\left(\frac{\pi a}{2}\right)}{\Gamma \left(\frac{\left(1+a\right)}{2}\right)\times a\times 2^{\left(\frac{a-1}{2}\right)}}\right)$$

Equations (6) and (7) depict the spiral form inside the search range, denoted by y and x, respectively. Equation (3) specifies this spiral form.

(6) $$y=r_{1}+UD_{1}\cos\left(-\omega D_{1}+\left(\frac{3\pi }{2}\right)\right)$$

(7) $$x=r_{1}+UD_{1}\sin\left(-\omega D_{1}+\left(\frac{3\pi }{2}\right)\right)$$

Variable $r_{1}$, over a predefined number of search iterations, takes values between 1 and 20. The constant values of ω and U are 0.005 and 0.00565, respectively. $D_{1}\in Z$ has a range from 1 to the dimension D of the search space.

#### 2.1.3. Expanded Exploitation

During the investigation phase, the Aquila bird meticulously examines the prey area before attacking with a low, slow fall. This strategy, sometimes referred to as expanded exploitation $x_{3}$, is represented mathematically in Equation (8).

(8) $$x_{3}^{\left(h+1\right)}=\left(x_{best}^{h}-x_{M}^{\left(h\right)}\right)\times \theta -rand+\left(\left(ub-lb\right)\times rand+lb\right)\times \rho$$

$\left(x_{3}^{\left(h+1\right)}\right)$, the result of Equation (8), represents the result for the subsequent iteration. In the $h^{th}$ iteration, $x_{best}\left(h\right)$ denotes the current best solution obtained, and $\left(x_{M}^{\left(h\right)}\right)$ denotes the average value of the current solution as determined by Equation (2). Variable “rand” is assigned a random number within the range of (0, 1), while tuning parameters θ and ρ are typically assigned values of 0.1 each. Symbols ub and lb represent the upper and lower bounds, respectively.

#### 2.1.4. Narrowed Exploitation

Aquila birds hunt by taking advantage of their prey’s unpredictable ground movement patterns to grab their prey directly. This hunting strategy serves as the basis for the constrained exploitation technique $\left(x_{4}^{\left(h\right)}\right)$ design, which is produced by Equation (9), which also yields the $h^{th}$ iteration of the following solution, denoted as $\left(x_{4}^{\left(h+1\right)}\right)$. Equation (10), which expresses the quality function J, was put out to provide a well-balanced search approach.

(9) $$x_{4}^{\left(h+1\right)}=J\times x_{best}^{\left(h\right)}-\left(P_{1}\times rand\times x_{1}^{\left(h\right)}\right)-P_{2}\times Levy\left(D\right)+rand\times P_{1}$$

Equations (11) and (12) are used to determine the mobility pattern for the Aquila’s prey tracking $\left(P_{1}\right)$ and the trajectory of an attack during an escape, from the beginning to the terminal point $\left(P_{2}\right)$. Both the maximum number of iterations H and the current iteration number h are used in the computations.

(10) $$J\left(h\right)=h^{\frac{2\times rand()-1}{{\left(1-H\right)}^{2}}}$$

(11) $$P_{1}=2\times rand-1$$

(12) $$P_{2}=2\times \left(1-\frac{h}{H}\right)$$

### 2.2. Concept of Dynamic Oppositional Learning (DOL)

The objectives of the optimization algorithms are to produce solutions, improve approximated solutions, and look for additional solutions inside the domain. The needs of tackling a complex problem cannot be met by the current solutions. Then, a variety of learning techniques are developed to improve optimization algorithms’ performance. Owing to its higher convergence capacity, the opposition-based learning (OBL) technique is the most frequently acknowledged among these learning systems. The following is an introduction to the definition of OBL [15]:

OBL is made up of the real number x∈R in the interval $x\in \left[a,b\right]$. Furthermore, the opposite number, $x^{OBL}$, is produced.

(13) $$x^{OBL}=a+b-x$$

Regarding a situation with several dimensions, the definition is demonstrated as follows: $x=\left(x_{1},x_{2},\ldots ,x_{D}\right)$ is a point in D dimensional coordinates if and only if $x_{1},x_{2},\ldots ,x_{D}\in R$ in the interval $\left[a_{i},b_{i}\right]$. As the iteration changes, the associated low and high bounds of the population are denoted by $a_{i}$ and $b_{i}$, respectively. In the meantime, the definition of the multidimensional opposite point is

(14) $$x_{i}^{OBL}=a_{i}+b_{i}-x_{i}$$

Even while the OBL method enhances the algorithm’s searching capabilities, it still has certain drawbacks, such being premature. Various variations of OBL have been proposed to enhance its performance. To expand the domain known as original notion of quasi-opposite-based learning (QOBL), for example, a quasi-opposite number is employed [17]. In the meantime, a quasi-reflection number is introduced in the interval between the present location and the center position in order to implement a quasi-reflection-based learning (QRBL) method [18].

Phase of Dynamic Opposite Learning: In addition to the OBL variations mentioned above, a novel learning approach called dynamic opposite learning operator (DOL) is used in this work. In order to enhance the TLBO algorithm’s performance, Xu et al. originally suggested the DOL method in [15]. When dealing with complex issues, the DOL is included to prevent the algorithm from being too young [19]. Furthermore, in an asymmetric and dynamic search environment, the DOL learning technique is a new variation of the opposition-based learning (OBL) strategy that aids in population learning from the opposite points [20,21].

#### 2.2.1. Dynamic Population Initialization

$x\in \left[a,b\right]$ was defined as the initial population in the initialization step. Additionally, $x^{OBL}$ is produced in the opposing domain. $x^{RO}\left(x^{RO}=rand\cdot x^{OBL},rand\in \left[0,1\right]\right)$ is introduced to replace $x^{OBL}$ in order to expand the searching space and convert the previous symmetric searching space into a dynamic asymmetric domain. The optimizer is then able to prevent prematurity by expanding the searching space. Therefore, in order to enhance the capacity to overcome local optima, a weighting factor $w_{d}$ is incorporated. This is how the mathematical model is displayed:

(15) $$x^{DOL}=x+w_{d}\cdot r_{1}\cdot \left(r_{2}\cdot x^{OBL}-x\right)$$

where $r_{2}\in \left[0,1\right]$ is a random parameter. When faced with a multifaceted goal, it manifests as follows:

(16) $$x_{ij}^{DOL}=x_{ij}+w_{d}\cdot r_{1}\cdot \left[r_{2}\cdot x_{ij}^{OBL}-x_{ij}\right]$$

where i=1, 2,…, N is the population size, j=1, 2,…, D is the dimension of an individual, $r_{1}$ and $r_{2}$ denote random numbers among $\left[0,1\right]$.

#### 2.2.2. Dynamic Population Jumping Process

In DOL, a jumping rate $\left(J_{r}\right)$ is used to update the population, and a positive weighting factor $\left(w_{d}\right)$ is employed to balance the capabilities of exploration and exploitation. The following is an implementation of the DOL operation procedure, provided that the selection probability is less than $J_{r}$.

(17) $$x_{ij}^{DOL}=x_{ij}+w_{d}\cdot r_{1}\cdot \left[r_{2}\cdot \left(a_{j}+b_{j}-x_{ij}\right)-x_{ij}\right]$$

where a random value $x_{ij}$ is produced as the starting populace; N is the population size; i is the $i^{th}$ solution; $x_{ij}^{DOL}$ is the population created by the DOL technique; j displays the dimension of $j^{th}$; two random parameters in $\left[0,1\right]$ are called $r_{1}$ and $r_{2}$; the weighting factor $w_{d}$ is set to 3; and the jumping rate $J_{r}$ is set at 1 by conducting sensitivity analysis as in Table 1.

### 2.3. Concept of Dynamic Random Walk (DRW)

Dynamic Random Walk (DRW) can be applied to the expanded exploration phase of the Aquila Optimizer metaheuristic algorithm to improve its exploration ability and help it escape local optima by the following equation:

(18) $$x=x_{best}+w\cdot r_{3}\cdot \left(r_{4}\cdot rwv-x_{best}\right)$$

where rwv, random walk vector, is provided by $rwv=r\left(1,D\right)-0.5$. Two random parameters in $\left[0,1\right]$ are called $r_{3}$ and $r_{4}$. DRW is incorporated into AO to improve its exploration ability. In the early stages of the optimization process, DRW is used to allow the search agents to explore a large search space.

## 3. Previous Work on AO and DOL

There is always room to enhance an algorithm by increasing and balancing the operators’ exploitation and exploration since the NFL theorem opposes the existence of an algorithm that is best suited for all optimization tasks. Plenty of work has been completed in the literature to improve the search efficiency in AO. These improvements include adjusting the algorithm’s parameters, including new movement strategies, and merging the algorithm with other optimization methods. The improved versions of AO can handle a large range of difficult real-world optimization problems better than the standard AO. The strategies used in AO are hybridization with NIOAs [22,23], oppositional-based learning [24], chaotic sequence [25], Levy flight-based strategy [26], Gauss map and crisscross operator [27], Niche Thought with Dispersed Chaotic Swarm [28], random learning mechanism and Nelder–Mead Simplex Search [29], wavelet mutation [30], Weighted Adaptive Searching Technique [31], Binay AO [32], and multi-objective AO [33].

DOL strategies are also used in many NIOAs to enhance their performance. First, they were introduced with Teaching–Learning-based Optimization [15], Grey Wolf Optimizer [34], Whale Optimization Algorithm [35], Antlion Optimizer [16], Bald Eagle Search Optimization [36], in the hybrid version of Aquila Optimizer, and Artificial Rabbits Optimization Algorithm [37], and the comprehensive survey with other algorithms can be found in the literature [14].

## 4. The Proposed DAO Algorithm

Two new features, DOL and DRW, are added to the original AO by the proposed DAO (Dynamic Random Walk and Dynamic Opposition Learning for Improving Aquila Optimizer) algorithm. The aim of DOL population generation is to provide diverse solutions to escape from stagnation, and DOL generation jumping helps in the exploitation ability of the algorithm and accelerates the speed of the algorithm. On the other hand, DRW will help the algorithm to improve its exploration ability. This overall approach will provide the perfect balance between exploration and exploitation and help the algorithm to escape from local optima. Let us examine this improvement working in more detail.

1. **Benefits of using DOL population initialization**

Compared to random initialization, the use of a dynamic opposition population initialization technique in Aquila Optimizer (AO) has various benefits that result in a more diverse solution pool:

(a) Random initialization limitations:
  Particularly for complex problems, random initialization might produce a population localized in a particular area of the search space, which restricts exploration and raises the possibility of becoming trapped in local optima.
(b) Initialization Based on Dynamic Opposition:
  For every randomly selected initial point, this method produces an “opposite” solution. With respect to a predetermined reference point (often the centre or limits), the opposing solution is located on the other side of the search area. This forces investigation in many places and produces wider initial dispersion of solutions.

The starting population is more diversified when opposition-based generation and random selection are combined. Because of this diversity, AO is able to investigate various regions of the search field right away. To prevent becoming overly biased in favor of the opposing alternatives, the strategy, nevertheless, maintains a healthy balance by retaining some randomly generated solutions. Overall, we can say that introducing DOL population initialization can help AO in the following ways:

(a) Increased exploration: AO can find promising regions throughout the whole search space by distributing the first solutions more widely.
(b) Decreased chance of local optima: AO is less likely to become stuck in solutions that are only effective in a small area because it starts from a variety of sites.
(c) Faster convergence: When multiple regions are investigated concurrently, a well-distributed population can converge more quickly to the global optimum.

2. **Benefits of using DOL generation jumping:**
  (a) Improved Exploration: Reintroducing exploration in later phases may result in the identification of more effective solutions.
  (b) Escape from Local Optima: AO is nudged away from regions that would not lead to the global optimum by jumping in opposition to underperforming individuals.
  (c) Fine-tuning: By investigating neighboring regions in the opposite direction, the leaps may discover somewhat better choices even if AO converges to a suitable solution.

3. **Benefits of using DRW in place of Aquila’s expanded exploration phase:**
  (a) Reduced Complexity: By doing away with the necessity to plan and carry out a specific extended exploration phase, DRW simplifies the algorithm as a whole.
  (b) Effective Exploration: Because of its intrinsic unpredictability, DRW can efficiently explore the search space and perhaps produce outcomes that are comparable to those of Aquila’s exploration stage.

In Algorithm 1, DOL Population Initialization and DOL Generation Jumping are used and DRW is used to swap out the expanded exploration of AO. Algorithm 1 illustrates the phases of this algorithm. In this, the parameter values are taken at their best regarding α, β of AO; weight $w_{d}$, jumping rate $J_{r}$ of DOL; and weight w of DRW for the rest of the paper.

**Figure 1:**
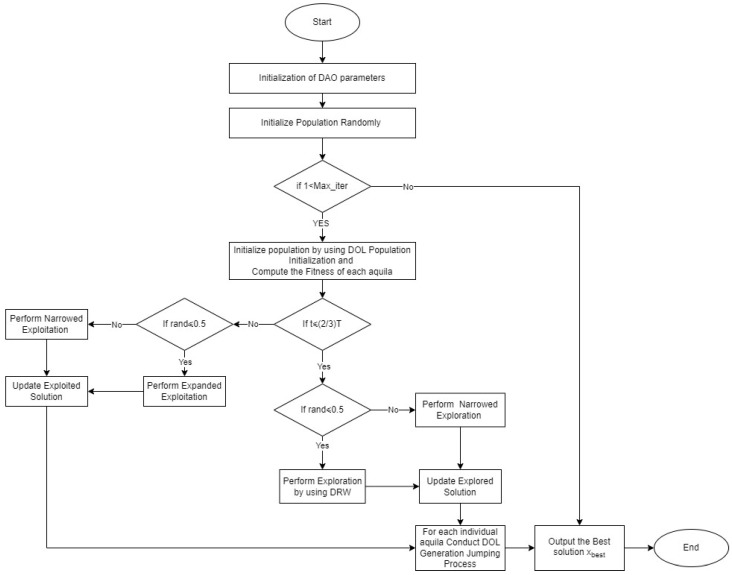
Flowchart of the proposed DAO algorithm.

Figure 1 also displays the algorithm DAO flowchart visualization.

**Algorithm 1** DAO Algorithm$\mathrm{Initialize}\;\mathrm{the}\;\mathrm{values}\;\mathrm{of}\;\mathrm{parameters}\;(\mathrm{nPop},\;\mathrm{nVar},\;\alpha ,\;\beta ,\;w,\;w_{d},\;J_{r}$, Max_iter, etc.)
Establish a random starting position.
Take the counter t=1
While (t < Max_iter), do
 Conduct DOL population initialization using Equation (16)
  Assess the early positions’ fitness.
 Verify Boundaries
  For (i = 1: nPop) do 
   Update of the existing solution’s mean value 
   Updated variables include $u,\;v,\;P_{1},\;P_{2},\;and\;Levy\left(D\right)$
       If $h\leq \left(\frac{2}{3}\right)\times Max\_iter$

          If rand≤0.5
           Apply DRW using Equation (18) 
          Else              Apply Narrowed Exploration by Equation (3) 
          End If 
       Else 
          If rand≤0.5
           Apply Expanded Exploitation by Equation (8) 
          Else           Apply Narrowed Exploitation by Equation (9) 
          End If
       End If
   Conduct the DOL population jumping process using Equation (17)
   Assess the fitness function. 
   Verify boundaries
  End for
t = t + 1
End while
Record best solution $\left(x_{best}\right)$


This section also displays DAO’s overall computational complexity. The initialization of the solutions, the computing of the fitness functions, and the updating of the solutions are the three steps that are often taken to ascertain the computational complexity of DAO. Let N represent the total number of solutions, and let $o\left(N\right)$ represent the computational complexity of the solutions’ initialization processes. The computational complexity of the updating processes for the solutions is $o\left(N\times D\right)+\;o\left(G\times \left(N\times D+N\times D\right)\right)$, where G is the total number of iterations and D is the size of the problem’s dimensions. These procedures entail updating the placements of each solution and looking for the best ones. Consequently, the overall computing complexity of the proposed DAO (Dynamic Opposition Learning and Dynamic Random Walk for Improving Aquila Optimizer) is $o\left(N\times D\right)+o\left(G\times \left(N\times D+N\times D\right)\right)=o\left(ND\left(1+2G\right)\right)$.

## 5. Experimental Settings

The algorithms used in the numerical trials include Aquila Optimizer (AO), Modified Aquila Optimizer (MAO) [38], Whale Optimization Algorithm (WOA) [6], Grasshopper Optimization Algorithm (GOA) [39], Reptile Search Algorithm (RSA) [5], and Brain Storm Optimization (BSO) [7]. On a computer with an Intel(R) Core (TM) i7-9750H processor running at 2.60 GHz and 16 GB of RAM, all algorithms were implemented in MATLAB R2021b.

The following five factors are used to assess DAO’s (Dynamic Opposition Learning and Dynamic Random Walk for Improving Aquila Optimizer) performance:

1. The optimization errors between the obtained and known real optimal values, average, and standard deviation. Since all objective functions include minimization, the best values—that is, the lowest mean values—are indicated in bold.
2. Non-parametric statistical tests to compare the *p*-value and the significance level = 0.05 between the compared technique and the suggested algorithm, such as the Wilcoxon rank sum test [40]. For both techniques, there is a significant difference when the *p*-value is less than 0.05. W/T/L indicates how many wins, ties, and losses the algorithm in question has suffered in contrast to its opponent.
3. The Friedman test is another non-parametric statistical test that is used [41,42]. The average optimization error values are used as test data. The method operates more efficiently with lower Friedman rank values. To make the minimal value stand out, it is bolded.
4. Bonferroni–Dunn’s diagram shows the differences in the rankings obtained for each algorithm at dimension 10 by showing the pairwise variances in ranks for each approach at each dimension. Pairwise disparities in rankings are calculated by subtracting the rank of one algorithm from the rank of another algorithm. In the graphic created by Bonferroni and Dunn, each bar denotes the average pairwise difference in ranks for a certain algorithm at a given dimension. Typically, different algorithms are represented by color-coded bars.
5. A clear visual depiction of the algorithm’s accuracy and convergence rate is offered via convergence graphs. If the improved algorithm deviates from the local answer, it explains why.

### 5.1. Competitive Algorithms Comparison on CEC2017 Benchmark Functions

Five competing algorithms are compared to gauge DAO’s efficiency and search performance: MAO (Modified Aquila Optimizer), AO (Aquila Optimizer), RSA (Reptile Search Algorithm), WOA (Whale Optimization Algorithm), and BSO (Brain Storm Optimization). The comparison is made on 29 benchmark functions from IEEE CEC2017 from the literature [43]. The population size (N) was fixed at 50 in each experiment. Maximum iteration is 500 and dimension is 10. The [−100, 100] range was chosen for the search. On each function, each algorithm was executed 30 times.

**Parameter Settings:** The algorithm’s performance depends on the parameter settings, particularly for DAO. In that instance, this part implements the sensitivity analysis of the parameters of DOL. Table 1 contains a detailed explanation of each parameter setting; the mean values are used to compare the results.

The weighting factor w and the jumping rate $J_{r}$ are set to 1–10 and 0.1–1 in the DAO algorithm, respectively. Here, in Table 1, only $J_{r}=0.3,\;\mathrm{and}\;1$ is taken because, at other points, the values are not favorable.

Test functions have been chosen for analysis from the literature [43], where F3 and F6 are multimodal functions, F18 is a hybrid function, F23 is a composition function, and, in order to assess performance, the means of the outcomes obtained by DAO are also shown in Table 1. In F3, F6, and F23, respectively, DAO performs better than other settings when w=3 and $J_{r}=1$. w=3 and $J_{r}=1$ are hence the best parameter settings, and DRW weight w=0.5 is taken from the literature [16]. Table 2 contains the parameter settings of the optimization algorithms used for comparison.

#### Analysis of IEEE CEC’17 Test Functions

- **Analysis of Unimodal and Multimodal Test Functions**

The mean and standard deviation of algorithms on twenty-nine unimodal, multimodal, and composition functions are displayed in Table 3. The function F1 is unimodal. The results show that, on one unimodal function, DAO outperforms the other algorithms. Moreover, it may be said that the DOL approach, which expands search spaces, has a higher chance of reaching the global optimum for its capacity for exploitation.

Multimodal functions like F3–F9 are used to confirm DAO’s exploring capabilities. The results in Table 3 demonstrate how well DAO performs in comparison to other algorithms, particularly on the F4, F5, F6, and F9 test functions.

- **Analysis of Hybrid and Composition Test Functions**

Hybrid functions are used to evaluate the algorithms by combining unimodal and multimodal functions in order to mimic real-world challenges. It may lead to subpar performance; however, balancing exploitation and exploration capability is important to deal with mixed tasks. Table 3 clearly illustrates the benefits of DAO on F12–F17, F20–F24, F26–F28, and F30, and the composition function indicates that DAO is still able to solve the problem to the same degree as other algorithms. Then, in many real-world scenarios, DAO may effectively balance the rate of convergence and the optimization solution.

The last line of Table 3 shows W/L/T (Win/Loss/Tie), Friedman rank, and CPU runtime. The W/L/T metric shows that DAO performs well on functions with 10 dimensions, outperforming AO, MAO, RSA, WOA, and BSO on 24, 29, 28, 28, and 27 functions, respectively. The Friedman rank of DAO is comparatively less than other MAs, and the CPU runtime of DAO, AO, MAO, RSA, WOA, and BSO is shown in the third-last line of Table 3. The results show that WOA takes much less time than other MAs.

Analysis of Convergence Graph

**Figure 2:**
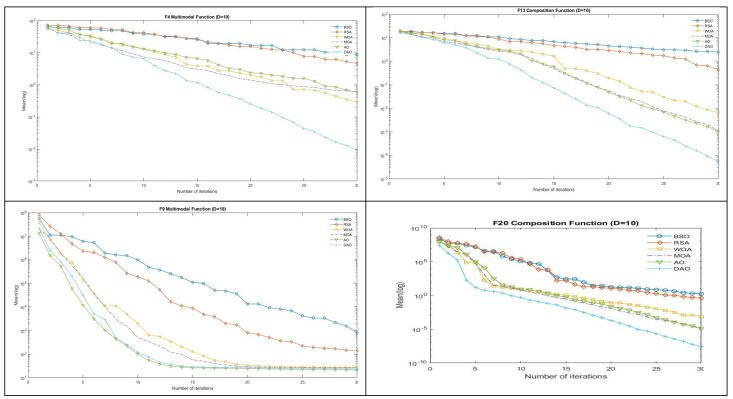
Convergence graphs of F4, F9, F13, and F20 CEC 2017 benchmark function.

Figure 2 displays the convergence graphs of the four functions, F4, F9, F13, and F20, where the mean optimizations generated by six algorithms on the IEEE CEC2017 functions with 10 dimensions are displayed. The vertical axis represents the log value of the mean optimizations, while the horizontal axis represents the number of iterations. Figure 2 makes it clear that the convergence speed is fast and that the DAO curves are the lowest. When compared to the original AO in the convergence graphs, DAO can find a better solution, exit local optimization, avoid premature convergence, improve the quality of the solution, and have high optimization efficiency.

Table 4 represents the Wilcoxon rank sum test results. The totals of ranks for positive and negative differences are represented by $\sum R^{+}$ and $\sum R^{-}$, respectively. When compared to other algorithms, DAO has a greater positive rank sum. Additionally, in the table, the corresponding z and *p* values are provided. The significant threshold of difference is α=0.05. This table shows that the performance of DAO is better than other original AO and other metaheuristic algorithms.

**Figure 3:**
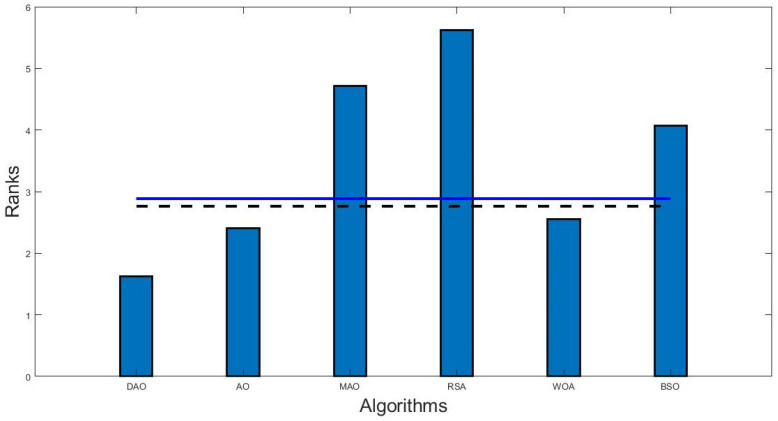
Bonferroni–Dunn bar chart for D = 10. The bar represents the rank of the correspondence algorithm, and horizontal cut lines show the significance level (here, ----- shows sig level at 0.1, and shows significance level at 0.05).

The Bonferroni–Dunn’s test [45] is used for the DAO algorithm to identify significant differences, and the results are shown in the last line of Table 4. Among all the other algorithms, DAO was found to have the lowest mean rank. The Bonferroni–Dunn graphic in Figure 3 shows the variation in ranks for each method at D = 10. In this figure, a horizontal cut line is drawn, which represents the threshold for the best-performing algorithm, the one with the lowest ranking bar. The height of this cut line is determined by adding the algorithm’s ranking. The Bonferroni–Dunn technique computed the equivalent CD for each α = 0.05 and α = 0.1. Algorithms with a rank bar higher than this line are deemed to perform worse than the control algorithm. As a result, it is evident from the use of the Bonferroni–Dunn technique that AO and WOA are substantially acceptable when compared with DAO.

### 5.2. Competitive Algorithms Comparison on CEC2019 Benchmark Functions

In Table 5, the list of the ten CEC2019 benchmark functions with their dimensions and search ranges is taken from the literature [46].

#### Analysis of IEEE CEC’19 Test Functions

DAO has been implemented on 10 CEC 2019 benchmark functions with 500 iterations and 50 population sizes for 30 independent runs. Its results are compared with AO, MAO, WOA, SSA, and GOA. The comparison has been performed through the mean and STD (standard deviation) values by the considered algorithms across the course of the functions, as reported in Table 6. Moreover, the Friedman mean rank values and W/L/T are involved in the table’s last lines (see Table 6). The results confirm the proposed DAO’s superiority in dealing with these challenging testbed functions as it is classified as the best algorithm for half of these functions.

**Figure 4:**
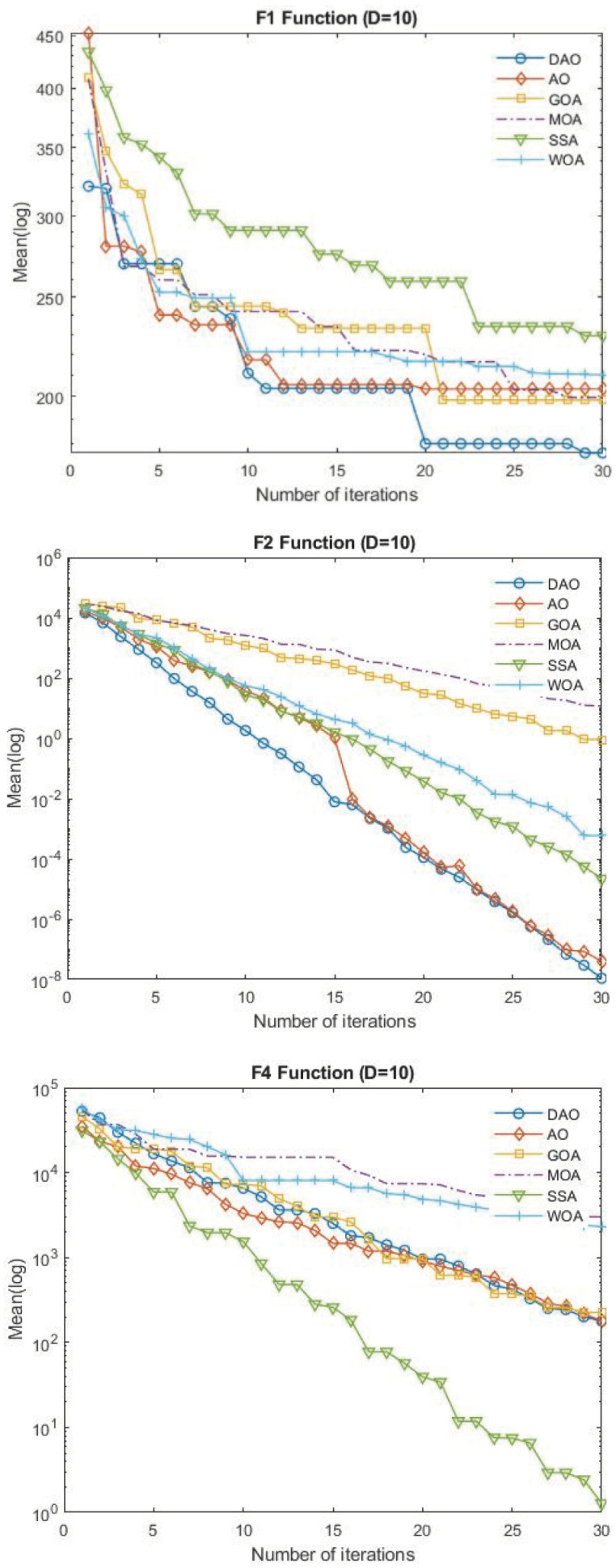

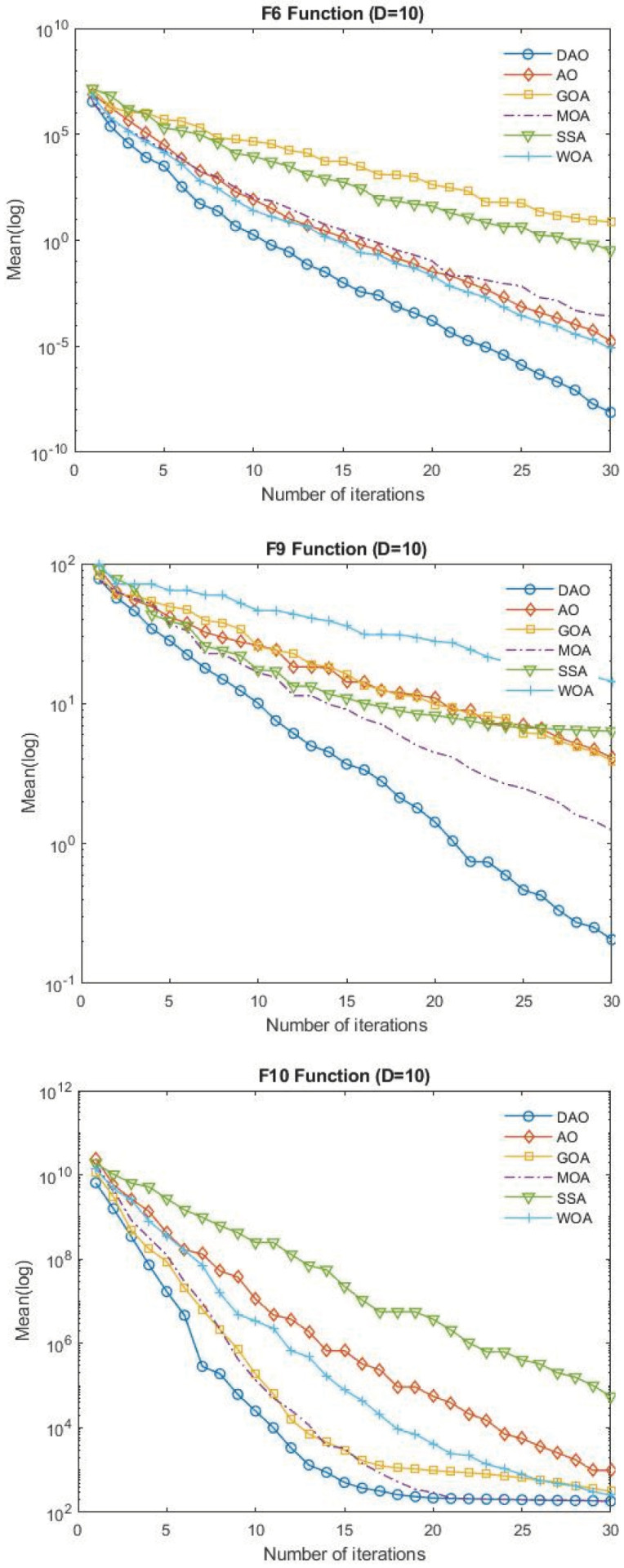
Convergence graphs of F1, F2, F4, F6, F9, and F10 CEC 2019 benchmark functions.

Meanwhile, AO succeeded for three functions, and MAO, WOA, SSA, and GOA for only one function out of this set. When it comes to the chain counterparts, DAO is positioned first in terms of sequence. The CPU runtime is mentioned in the last line of Table 6, which shows WOA taking much less time than the other algorithms. The convergence curves of Figure 4 show the efficiency of DAO in converging for high qualified solutions with significant convergence speed, as exhibited in F2, F6, F7, and F9.

Figure 4 shows the convergence capacity of six algorithms on test functions. The average fitness value is displayed as the “Mean”. Because of its exceptional exploration capabilities, DAO converges quickly with iterative computation, as illustrated in the figures. Regarding the trend that is gradually convergent, this is because the DOL technique is capable of being exploited.

Table 7 represents the Wilcoxon rank sum test results. The totals of ranks for positive and negative differences are represented by $\sum R^{+}$ and $\sum R^{-}$, respectively. When compared to other algorithms, DAO has a greater positive rank sum in most of the cases. Additionally, in the table, the corresponding z and *p* values are provided. The significant threshold of difference is α=0.05. This table shows that the performance of DAO is equivalently acceptable when compared to other metaheuristic algorithms.

**Figure 5:**
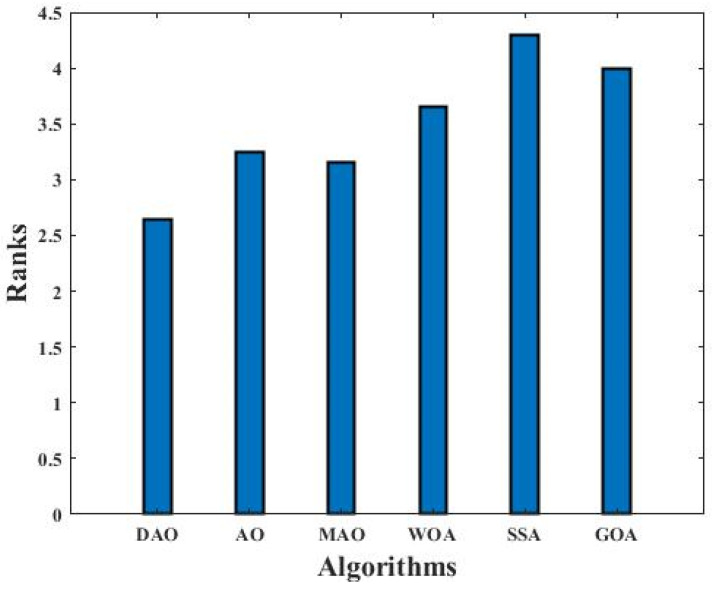
Bonferroni–Dunn bar chart for D = 10. The bar represents the rank of the correspondence algorithm.

Bonferroni–Dunn’s test is used for the DAO algorithm to identify significant differences, and the results are shown in the last line of Table 7. Among all the other algorithms, DAO was found to have the lowest mean rank. The Bonferroni–Dunn graphic in Figure 5 shows the variation in ranks for each method at D = 10. In this figure, the smallest bar will show the best-performing algorithm, or the one with the lowest ranking bar. Algorithms with a higher rank bar are deemed to perform worse than the control algorithm. As a result, it is evident from the use of the Bonferroni–Dunn technique that DAO is also performing well when compared with other metaheuristic algorithms, and the worst performance is from the SSA algorithm.

## 6. DAO for Engineering Design Problems

Three relevant engineering benchmarks are used in this section to confirm that DAO improves when tackling real-world problems: problem of cantilever beam design (CBD), welded beam design problem (WBD), and pressure vessel design (PVD) problem and work. Thirty independent runs of each problem were carried out in order to examine the statistical features of the outcomes, and all the parameters are taken at their best.

### 6.1. CBD Problem

The goal of the CBD problem is to minimize a cantilever beam’s weight while accounting for the vertical displacement constraint. There are five hollow square blocks, and each of the five side length values $z_{1},\;z_{2},\;z_{3},\;z_{4},\;z_{5}$ needs to be optimized [47]. The following is an explanation of the mathematical model:

Consider

$$z=\left[z_{1}\;z_{2}\;z_{3}\;z_{4}\;z_{5}\right]$$

Minimize

$$f(z)=0.6224(z_{1}+z_{2}+z_{3}+z_{4}+z_{5}),$$

Subject to

$$p(z)=\frac{60}{z_{1}^{3}}+\frac{27}{z_{2}^{3}}+\frac{19}{z_{3}^{3}}+\frac{7}{z_{4}^{3}}+\frac{1}{z_{5}^{3}}-1\leq 0$$

Variable range is

$$0.01\leq z_{1},z_{2},z_{3},z_{4},z_{5}\leq 100$$

Table 8 displays the results of the CBD problem compared with six different MAs, such as COA, AO, GWO, ROA, WOA, and SCA. The results indicate that the proposed algorithm DAO is able to provide better results than other state-of-the-art algorithms. Thus, DAO is the optimal method for addressing the CBD problem. CPU runtime of the given set of algorithms is calculated, which shows that WOA takes very little time to compute the CBD problem.

### 6.2. WBD Problem

The goal of the WBD challenge is to reduce the cost of manufacturing a welded beam [9]. The optimization parameters include thickness (H), height ($T_{T}$), length of the clamping bar (L), and thickness ($B_{B}$). It is important to take into account seven limitations. The optimization model can be stated as follows:

Consider

$$z=\left[z_{1}\;z_{2}\;z_{3}\;z_{4}\right]=\left[H\;L\;T_{T}\;B_{B}\right]$$

Minimize

$$f(z)=1.10471z_{1}^{2}z_{z}+0.04811z_{3}z_{4}(14.0+z_{2})$$

Subject to the constraint,

$$\begin{array}{l} p_{1}(z)=\tau \left(z\right)-\tau_{\max}\leq 0,\\ p_{2}(z)=\sigma \left(z\right)-\sigma_{\max}\leq 0,\\ p_{3}(z)=\delta \left(z\right)-\delta_{\max}\leq 0,\\ p_{4}(z)=z_{1}-z_{4}\leq 0,\\ p_{5}(z)=P-P_{c}\left(z\right)\leq 0,\\ p_{6}(z)=0.125-z_{1}\leq 0,\\ p_{7}(z)=1.10471z_{1}^{2}+0.04811z_{3}z_{4}\left(14+z_{2}\right)-5\leq 0 \end{array}$$

Variable range

$$\begin{array}{l} 0.1\leq z_{1}\leq 2,\\ 0.1\leq z_{2}\leq 10,\\ 0.1\leq z_{3}\leq 10,\\ 0.1\leq z_{4}\leq 2 \end{array}$$

where

$$\begin{array}{l} \tau \left(z\right)=\sqrt{{\left(\tau^{\prime }\right)}^{2}+2\tau^{\prime }\tau^{\prime \prime }\frac{z_{2}}{2R}+{\left(\tau^{\prime \prime }\right)}^{2}},\\ \tau^{\prime }=\frac{p}{\sqrt{2z_{1}z_{2}}},\;\tau^{\prime \prime }=\frac{MR}{J} \end{array}$$

$$\begin{array}{l} M=P\left(L+\frac{z_{2}}{2}\right),\\ R=\sqrt{\frac{z_{2}^{2}}{4}+{\left(\frac{z_{1}+z_{3}}{2}\right)}^{2}},\\ J=2\left\{\sqrt{z_{1}z_{2}}\left[\frac{z_{2}^{2}}{4}+{\left(\frac{z_{1}+z_{3}}{2}\right)}^{2}\right]\right\},\\ \sigma \left(z\right)=\frac{6PL}{z_{4}z_{3}^{2}},\delta \left(z\right)=\frac{6PL^{3}}{Ez_{3}^{2}z_{4}}\\ P_{c}\left(z\right)=\frac{\sqrt[{4.013E}]{\frac{z_{3}^{2}z_{4}^{6}}{36}}}{L^{2}}\left(1-\frac{z_{3}}{2L}\sqrt{\frac{E}{4G}}\right), \end{array}$$

$$\begin{array}{l} P=6000\;\mathrm{lb},\;L=14\;\mathrm{in}.,\;\delta_{\max}=0.25\;\mathrm{in}.,\\ E=30\times 1^{6}\;\mathrm{psi},\;G=12\times 10^{6}\;\mathrm{psi},\\ \tau_{\max}=13600\;\mathrm{psi},\;\sigma_{\max}=30000\;\mathrm{psi} \end{array}$$

Table 9 reports the outcomes of the WBD problem. It is clear that DAO is not able to provide a better solution than other algorithms. However, with the exception of AO, DAO also has a very close value to provide an optimal result. This suggests that DAO is a stable and effective solution to the WBD problem. CPU runtime of the given set of algorithms is calculated, which shows that WOA takes very little time to compute the WBD problem.

### 6.3. PVD Problem

The PVD problem, a classical and representative optimization issue in engineering, is typically employed to verify the efficacy of optimization techniques. Its goal is to reduce a tension/compression spring’s cost [41]. The design parameters are thickness of the shell $T_{S}$, thickness of the head $T_{H}$, inner radius r, and the length of the cylindrical shell $L_{CS}$. The following is the expression for the mathematical formulation [47]:

Consider

$$z=[z_{1}\;z_{2}\;z_{3}\;z_{4}]=\left[T_{S}\;T_{H}\;r\;L_{CS}\right]$$

Minimize

$$f(z)=0.6224z_{1}z_{3}z_{4}+1.7781z_{2}z_{3}^{2}+3.1661z_{1}^{2}z_{4}+19.84z_{1}^{2}z_{3},$$

Subject to

$$\begin{array}{c} p_{1}(z)=-z_{1}+0.0193z_{3}\leq 0,\\ p_{2}(z)=-z_{3}+0.00954z_{3}\leq 0, \end{array}$$

$$p_{3}(z)=-\pi z_{3}^{2}z_{4}-\frac{4}{3}\pi z_{3}^{3}+1296000\leq 0,$$

$$p_{4}(z)=z_{4}-240\leq 0$$

Variable range is

$$\left\{\begin{array}{c} 0\leq z_{1}\leq 99,\\ 0\leq z_{2}\leq 99,\\ 10\leq z_{3}\leq 200,\\ 10\leq z_{4}\leq 200 \end{array}\right.$$

Table 10′s results for the TSD problem demonstrate that ROA is the optimal method for solving it, followed by COA and DAO, but we can say that DAO is a competitive and stable solution. CPU runtime of the given set of algorithms is calculated, which shows that WOA takes very little time to compute the PVD problem.

The outcomes of three classic engineering challenges are shown in this section, demonstrating how well and consistently DAO performs when handling real-world issues. In particular, DAO performs noticeably better than the AO algorithm.

## 7. Conclusions

In order to replace the expanded exploration regarding AO, this study has proposed a low-complexity DRW method that strikes a fair balance between exploitation and exploration. The aim of this technique is to increase computational efficiency and to avoid stagnation. Moreover, to achieve a balance between exploration and exploitation, the DOL technique is introduced. The CPU runtime clearly shows Aquila Optimizer’s computing efficiency. Then, the results obtained from the benchmark functions of CEC 2017 and CEC 2019 demonstrate its superiority. Furthermore, the convergence graphs, the Wilcoxon rank sum tests, the Friedman test, and the Bonferroni test show its accessibility. Then, it is also applied to real-world structural engineering design problems, which provides better results than AO. All these results show that the DRW and DOL approaches provide great additions to AO. DAO performs far better than AO as well as compared to most of the other MAs.

## 8. Future Scope

DAO could be applied in additional real-world applications given its great performance. Additionally, other optimization jobs including image processing, cloud and fog computing, and others could use the DAO optimization method.

## Figures and Tables

**Table 1:** The sensitivity analysis of w and $J_{r}$.

w	Mean							
	F3		F6		F18		F23	
	$J_{r}\;=\;0.3$	$J_{r}\;=\;1$	$J_{r}\;=\;0.3$	$J_{r}\;=\;1$	$J_{r}\;=\;0.3$	$J_{r}\;=\;1$	$J_{r}\;=\;0.3$	$J_{r}\;=\;1$
1	6.582 × 10^3^	6.859 × 10^3^	4.251 × 10^1^	4.839 × 10^1^	2.387 × 10^5^	**1.457 × 10^5^**	4.256 × 10^2^	4.340 × 10^2^
2	6.502 × 10^3^	5.187 × 10^3^	4.216 × 10^1^	3.776 × 10^1^	3.041 × 10^5^	1.567 × 10^5^	4.101 × 10^2^	3.865 × 10^2^
3	6.750 × 10^3^	**4.022 × 10^3^**	4.469 × 10^1^	**3.675 × 10^1^**	2.224 × 10^6^	6.025 × 10^5^	3.982 × 10^2^	**3.818 × 10^2^**
4	8.326 × 10^3^	5.279 × 10^3^	4.476 × 10^1^	3.885 × 10^1^	1.045 × 10^6^	6.649 × 10^5^	4.082 × 10^2^	3.868 × 10^2^
5	8.613 × 10^3^	4.975 × 10^3^	4.468 × 10^1^	3.908 × 10^1^	4.604 × 10^6^	1.106 × 10^6^	4.109 × 10^2^	3.888 × 10^2^
6	9.230 × 10^3^	5.749 × 10^3^	4.886 × 10^1^	4.031 × 10^1^	3.238 × 10^6^	2.014 × 10^6^	4.166 × 10^2^	4.025 × 10^2^
7	9.451 × 10^3^	7.962 × 10^3^	5.108 × 10^1^	4.931 × 10^1^	5.852 × 10^6^	5.806 × 10^6^	4.192 × 10^2^	4.218 × 10^2^
8	1.031 × 10^4^	8.989 × 10^3^	5.228 × 10^1^	4.922 × 10^1^	3.966 × 10^6^	1.155 × 10^7^	4.223 × 10^2^	4.357 × 10^2^
9	9.738 × 10^3^	1.003 × 10^4^	5.263 × 10^1^	5.246 × 10^1^	1.043 × 10^7^	1.255 × 10^7^	4.181 × 10^2^	4.472 × 10^2^
10	1.087 × 10^4^	1.108 × 10^4^	5.446 × 10^1^	5.205 × 10^1^	2.989 × 10^7^	3.067 × 10^7^	4.311 × 10^2^	4.574 × 10^2^

Note: bold is used to indicate the best result.

**Table 2:** Parameter settings of optimization algorithms.

Algorithm	Parameters
DAO	$U=0.00565,r=10,\omega =0.05,\alpha =0.1,\beta =0.1,P_{1}\in \left[-1,1\right],P_{2}=\left[2,0\right],w=0.5,w_{d}=3,J_{r}=1$
AO [9]	$U=0.00565,r=10,\omega =0.05,\alpha =0.1,\beta =0.1,P_{1}\in \left[-1,1\right],P_{2}=\left[2,0\right]$
MAO [38]	$U=0.00565,r=10,\omega =0.05,\alpha =0.1,\beta =0.1,P_{1}\in \left[-1,1\right],P_{2}=\left[2,0\right]$
SSA [44]	v=0
WOA [6]	$w_{1}=\left[2,0\right],w_{2}=\left[-1,-2\right],v=1$
RSA [5]	$a=\left[2,0\right]$
GOA [39]	l=1.5, f=0.5
BSO [7]	$m=5,\;p_{a}=0.2,\;p_{b}=0.8,\;p_{b_{1}}=0.4,\;p_{c}=0.5$

**Table 3:** Mean and standard deviation (STD) obtained from objective function by standard AO, the proposed algorithm DAO, and other metaheuristic algorithms for 10-dimensional CEC 2017 benchmark functions.

Function	DAO	AO	MAO	RSA	WOA	BSO
F1 Mean STD	**8.388 × 10^8^** 4.709 × 10^8^	9.239 × 10^8^ 6.512 × 10^6^	2.159 × 10^10^ 4.981 × 10^9^	5.38 × 10^10^ 9.29 × 10^9^	9.784 × 10^8^ 7.462 × 10^6^	9.410 × 10^9^ 2.501 × 10^3^
F3 Mean STD	4.291 × 10^3^ 1.381 × 10^3^	8.809 × 10^2^ 5.485 × 10^2^	2.363 × 10^5^ 4.971 × 10^4^	7.42 × 10^4^ 5.50 × 10^3^	3.663 × 10^3^ 3.232 × 10^3^	**3.001 × 10^1^** 1.710 × 10^2^
F4 Mean STD	**7.619 × 10^1^** 4.001 × 10^1^	2.085 × 10^2^ 2.512 × 10^2^	2.527 × 10^3^ 1.304 × 10^3^	1.45 × 10^4^ 4.56 × 10^3^	9.451 × 10^1^ 1.923 × 10^1^	9.495 × 10^2^ 2.001 × 10^1^
F5 Mean STD	**6.359 × 10^1^** 1.584 × 10^1^	7.125 × 10^1^ 1.066 × 10^1^	1.508 × 10^2^ 2.758 × 10^1^	3.89 × 10^2^ 3.30 × 10^1^	8.408 × 10^1^ 2.096 × 10^1^	2.038 × 10^2^ 4.101 × 10^1^
F6 Mean STD	**3.523 × 10^1^** 8.702 × 10^0^	7.745 × 10^1^ 6.053 × 10^0^	9.271 × 10^1^ 1.745 × 10^1^	8.63 × 10^1^ 7.46 × 10^0^	3.627 × 10^1^ 1.012 × 10^1^	5.316 × 10^1^ 6.414 × 10^0^
F7 Mean STD	8.615 × 10^1^ 2.031 × 10^1^	**5.545 × 10^1^** 1.931 × 10^1^	4.585 × 10^2^ 9.379 × 10^1^	6.72 × 10^2^ 6.73 × 10^1^	7.470 × 10^1^ 2.151 × 10^1^	5.110 × 10^2^ 1.011 × 10^2^
F8 Mean STD	3.211 × 10^1^ 6.033 × 10^0^	**2.408 × 10^1^** 6.884 × 10^0^	1.369 × 10^2^ 1.922 × 10^1^	3.11 × 10^2^ 2.80 × 10^1^	4.291 × 10^1^ 1.767 × 10^1^	1.451 × 10^2^ 3.211 × 10^1^
F9 Mean STD	**2.773 × 10^2^** 1.571 × 10^2^	3.135 × 10^2^ 6.321 × 10^1^	4.114 × 10^3^ 1.082 × 10^3^	8.53 × 10^3^ 1.19 × 10^3^	5.919 × 10^2^ 3.820 × 10^2^	3.411 × 10^3^ 6.754 × 10^2^
F10Mean STD	1.451 × 10^3^ 3.124 × 10^2^	**9.451 × 10^2^** 2.686 × 10^2^	2.726 × 10^3^ 2.296 × 10^2^	7.02 × 10^3^ 3.59 × 10^2^	1.181 × 10^3^ 2.751 × 10^2^	4.211 × 10^3^ 6.081 × 10^2^
F11Mean STD	4.201 × 10^2^ 4.743 × 10^2^	**1.078 × 10^2^** 5.818 × 10^1^	2.604 × 10^4^ 2.681 × 10^4^	7.77 × 10^3^ 2.80 × 10^3^	1.417 × 10^2^ 8.465 × 10^1^	1.378 × 10^2^ 4.511 × 10^1^
F12Mean STD	**5.697 × 10^6^** 5.285 × 10^6^	7.862 × 10^6^ 3.363 × 10^6^	2.784 × 10^9^ 1.640 × 10^9^	1.70 × 10^10^ 4.36 × 10^9^	7.279 × 10^6^ 5.117 × 10^6^	9.614 × 10^7^ 8.094 × 10^5^
F13Mean STD	**2.549 × 10^5^** 6.824 × 10^5^	2.465 × 10^5^ 1.528 × 10^4^	3.020 × 10^8^ 3.011 × 10^8^	1.18 × 10^10^ 4.90 × 10^9^	1.437 × 10^6^ 1.177 × 10^4^	5.216 × 10^7^ 2.340 × 10^4^
F14Mean STD	**5.424 × 10^3^** 8.248 × 10^3^	6.334 × 10^4^ 8.016 × 10^2^	7.503 × 10^6^ 1.063 × 10^7^	3.07 × 10^6^ 3.58 × 10^6^	7.307 × 10^3^ 1.500 × 10^3^	4.170 × 10^5^ 3.152 × 10^3^
F15Mean STD	**6.293 × 10^3^** 3.375 × 10^3^	9.332 × 10^3^ 2.839 × 10^3^	2.148 × 10^7^ 2.908 × 10^7^	6.73 × 10^8^ 5.74 × 10^8^	6.416 × 10^3^ 5.063 × 10^3^	3.112 × 10^4^ 2.122 × 10^4^
F16Mean STD	**3.248 × 10^2^** 1.027 × 10^2^	9.535 × 10^2^ 1.114 × 10^2^	1.178 × 10^3^ 2.349 × 10^2^	3.89 × 10^3^ 6.86 × 10^2^	3.329 × 10^2^ 1.440 × 10^2^	1.504 × 10^3^ 3.314 × 10^2^
F17Mean STD	**8.911 × 10^1^** 2.286 × 10^1^	9.589 × 10^1^ 1.871 × 10^1^	6.631 × 10^2^ 1.916 × 10^2^	5.30 × 10^3^ 6.86 × 10^3^	1.033 × 10^2^ 5.087 × 10^1^	8.120 × 10^2^ 2.401 × 10^2^
F18Mean STD	2.407 × 10^5^ 3.197 × 10^5^	2.153 × 10^4^ 1.184 × 10^4^	6.274 × 10^8^ 6.430 × 10^8^	3.27 × 10^7^ 3.07 × 10^7^	**1.946 × 10^4^** 1.111 × 10^4^	1.120 × 10^5^ 1.001 × 10^5^
F19Mean STD	3.203 × 10^4^ 4.889 × 10^4^	1.436 × 10^4^ 2.225 × 10^4^	6.471 × 10^7^ 8.754 × 10^7^	**1.32 × 10^4^** 1.69 × 10^9^	6.597 × 10^4^ 9.665 × 10^4^	1.301 × 10^5^ 5.361 × 10^4^
F20Mean STD	**1.701 × 10^2^** 5.579 × 10^1^	2.153 × 10^2^ 4.716 × 10^1^	5.419 × 10^2^ 1.330 × 10^2^	8.63 × 10^2^ 1.42 × 10^2^	1.854 × 10^2^ 7.896 × 10^1^	7.219 × 10^2^ 2.015 × 10^2^
F21Mean STD	**2.299 × 10^2^** 5.293 × 10^1^	2.967 × 10^2^ 4.681 × 10^1^	3.375 × 10^2^ 3.148 × 10^1^	6.43 × 10^2^ 4.26 × 10^1^	2.310 × 10^2^ 5.171 × 10^1^	4.004 × 10^2^ 4.051 × 10^1^
F22Mean STD	**1.758 × 10^2^** 5.337 × 10^1^	2.091 × 10^2^ 1.524 × 10^1^	1.798 × 10^3^ 5.866 × 10^2^	5.25 × 10^3^ 1.01 × 10^3^	1.831 × 10^2^ 2.703 × 10^2^	4.001 × 10^3^ 1.701 × 10^3^
F23Mean STD	**3.843 × 10^2^** 2.354 × 10^1^	5.412 × 10^2^ 1.313 × 10^1^	5.423 × 10^2^ 6.781 × 10^1^	1.04 × 10^3^ 1.08 × 10^2^	3.976 × 10^2^ 2.060 × 10^1^	9.991 × 10^2^ 1.013 × 10^2^
F24Mean STD	**3.145 × 10^2^** 1.416 × 10^1^	3.437 × 10^2^ 8.266 × 10^1^	5.939 × 10^2^ 7.436 × 10^1^	1.17 × 10^3^ 2.45 × 10^2^	3.870 × 10^2^ 2.521 × 10^1^	1.004 × 10^3^ 9.711 × 10^1^
F25Mean STD	4.776 × 10^2^ 4.645 × 10^1^	7.949 × 10^2^ 3.036 × 10^1^	1.988 × 10^3^ 7.365 × 10^2^	2.22 × 10^3^ 8.61 × 10^2^	5.651 × 10^2^ 3.538 × 10^1^	**4.101 × 10^2^** 9.110 × 10^0^
F26Mean STD	**6.408 × 10^2^** 3.023 × 10^2^	9.175 × 10^2^ 1.623 × 10^2^	2.348 × 10^3^ 3.639 × 10^2^	7.93 × 10^3^ 1.12 × 10^3^	9.465 × 10^2^ 6.068 × 10^2^	5.832 × 10^3^ 1.112 × 10^3^
F27Mean STD	**4.467 × 10^2^** 4.845 × 10^1^	6.041 × 10^2^ 8.332 × 10^0^	7.303 × 10^2^ 1.222 × 10^2^	9.41 × 10^2^ 2.31 × 10^2^	5.379 × 10^2^ 3.300 × 10^1^	1.204 × 10^3^ 2.510 × 10^2^
F28Mean STD	**4.913 × 10^2^** 6.521 × 10^0^	5.965 × 10^2^ 9.938 × 10^1^	1.323 × 10^3^ 2.043 × 10^2^	3.98 × 10^3^ 8.85 × 10^2^	6.153 × 10^2^ 1.794 × 10^2^	5.854 × 10^2^ 5.120 × 10^1^
F29Mean STD	4.026 × 10^2^ 6.510 × 10^1^	**3.429 × 10^2^** 5.123 × 10^1^	1.070 × 10^3^ 2.163 × 10^2^	4.14 × 10^3^ 1.61 × 10^3^	4.614 × 10^2^ 8.636 × 10^1^	1.520 × 10^3^ 3.701 × 10^2^
F30Mean STD	**3.891 × 10^4^** 8.456 × 10^4^	6.647 × 10^5^ 7.482 × 10^4^	1.451 × 10^8^ 1.052 × 10^8^	2.24 × 10^8^ 9.25 × 10^7^	7.597 × 10^6^ 9.042 × 10^5^	5.371 × 10^5^ 3.104 × 10^5^
(W/L/T) Rank CPU Runtime	20/9/0 **1.62** 3.25 × 10^4^	5/24/0 2.41 2.10 × 10^4^	0/29/0 4.72 1.29 × 10^4^	1/28/0 5.62 5.11 × 10^4^	1/28/0 2.55 **4.10 × 10^3^**	2/27/0 4.07 1.29 × 10^4^

Note: bold is used to indicate the best result.

**Table 4:** Summary of non-parametric statistical results by Wilcoxon test and Bonferroni–Dunn test.

Algorithms		$\sum R^{+}$	$\sum R^{-}$	*z*-Value	*p*-Value	Sign
DAO vs.	AO	21	8	2.022	0.043	=
	MAO	29	0	4.703	0.000	+
	RSA	28	1	4.249	0.000	+
	WOA	24	5	2.757	0.006	=
	BSO	25	4	3.557	0.000	+
CD value at α=0.1	1.1428			CD value at α=0.05	1.2656	

**Table 5:** List of 10 benchmark functions of CEC2019 with dimensions and search range.

Func. No.	Functions	Dim	Search Range
F1	Storn’s Chebyshev Polynomial Fitting Problem	9	[−8192, 8192]
F2	Inverse Hilbert Matrix Problem	16	[−16,384, 16,384]
F3	Lennard-Jones Minimum Energy Cluster	18	[−4, 4]
F4	Rastrigin’s Function	10	[−100, 100]
F5	Griewangk’s Function	10	[−100, 100]
F6	Weierstrass Function	10	[−100, 100]
F7	Modified Schwefel’s Function	10	[−100, 100]
F8	Expanded Schaffer’s F6 Function	10	[−100, 100]
F9	Happy Cat Function	10	[−100, 100]
F10	Ackley Function	10	[−100, 100]

**Table 6:** Mean and standard deviation (STD) obtained from objective function by standard AO, the proposed algorithm DAO, and other metaheuristic algorithms for 10-dimensional CEC 2019 benchmark functions.

Function	DAO	AO	MAO	WOA	SSA	GOA
F1 Mean STD	**9.900 × 10^1^** 0.000 × 10^0^	**9.900 × 10^1^** 2.053 × 10^−8^	1.235 × 10^9^ 7.355 × 10^8^	6.784 × 10^6^ 7.462 × 10^6^	7.324 × 10^9^ 3.483 × 10^9^	1.320 × 10^10^ 1.541 × 10^10^
F2 Mean STD	**1.950 × 10^2^** 0.000 × 10^0^	**1.950 × 10^2^** 0.000 × 10^0^	2.825 × 10^4^ 7.301 × 10^3^	7.663 × 10^2^ 8.7317 × 10^2^	2.001 × 10^2^ 2.079 × 10^−2^	1.739 × 10^3^ 4.084 × 10^2^
F3 Mean STD	2.948 × 10^2^ 1.321 × 10^0^	2.937 × 10^2^ 1.863 × 10^0^	2.862 × 10^2^ 4.426 × 10^−1^	2.951 × 10^2^ 1.923 × 10^0^	2.970 × 10^2^ 1.776 × 10^−15^	**2.270 × 10^2^** 8.188 × 10^−12^
F4 Mean STD	3.441 × 10^2^ 1.376 × 10^1^	3.683 × 10^2^ 9.697 × 10^0^	2.445 × 10^2^ 2.501 × 10^1^	3.498 × 10^2^ 2.496 × 10^1^	**3.423 × 10^1^** 1.077 × 10^1^	3.286 × 10^2^ 1.971 × 10^1^
F5 Mean STD	4.929 × 10^2^ 4.413 × 10^0^	4.981 × 10^2^ 1.826 × 10^−1^	**3.059 × 10^2^** 4.894 × 10^1^	4.977 × 10^2^ 4.591 × 10^−1^	5.486 × 10^2^ 8.533 × 10^−1^	8.484 × 10^2^ 8.763 × 10^−1^
F6 Mean STD	**5.918 × 10^2^** 1.787 × 10^0^	5.944 × 10^2^ 1.440 × 10^0^	5.999 × 10^2^ 9.224 × 10^−1^	5.919 × 10^2^ 1.751 × 10^0^	5.986 × 10^2^ 8.533 × 10^−1^	8.484 × 10^2^ 8.763 × 10^1^
F7 Mean STD	7.152 × 10^2^ 2.553 × 10^2^	**3.011 × 10^2^** 2.936 × 10^2^	2.217 × 10^3^ 2.924 × 10^2^	7.640 × 10^2^ 3.001 × 10^2^	4.728 × 10^2^ 9.776 × 10^−1^	5.007 × 10^2^ 2.191 × 10^2^
F8 Mean STD	7.953 × 10^2^ 1.998 × 10^−1^	8.957 × 10^2^ 3.015 × 10^−1^	7.644 × 10^2^ 2.387 × 10^−1^	**5.953 × 10^2^** 3.216 × 10^−1^	9.088 × 10^2^ 6.135 × 10^−1^	8.587 × 10^2^ 4.300 × 10^−1^
F9 Mean STD	**8.985 × 10^2^** 1.639 × 10^−1^	9.365 × 10^2^ 1.427 × 10^−1^	8.993 × 10^3^ 8.679 × 10^−1^	8.985 × 10^3^ 2.006 × 10^−1^	2.416 × 10^3^ 5.956 × 10^−1^	9.664 × 10^2^ 1.827 × 10^−1^
F10 Mean STD	**9.785 × 10^2^** 7.688 × 10^−1^	9.996 × 10^2^ 4.637 × 10^0^	9.852 × 10^2^ 1.350 × 10^−1^	9.953 × 10^2^ 1.330 × 10^−1^	2.101 × 10^3^ 3.562 × 10^1^	9.923 × 10^2^ 3.718 × 10^−4^
(W/L/T) Rank CPU Runtime	5/5/2 **2.65** 3.11 × 10^4^	3/7/2 3.25 3.02 × 10^4^	1/9/0 3.15 2.16 × 10^4^	1/9/0 3.65 5.02 × 10^4^	1/9/0 4.30 **4.22 × 10^3^**	1/9/0 4.00 1.26 × 10^4^

Note: bold is used to indicate the best result.

**Table 7:** Summary of non-parametric statistical results obtained from Wilcoxon test and Bonferroni–Dunn test.

Algorithms		ΣR^+^	ΣR^−^	z-Value	*p*-Value	Sign
DAO vs.	AO	6	2	1.260	0.208	=
	MAO	5	5	0.866	0.386	=
	WOA	6	3	1.599	0.110	=
	MPA	8	2	1.478	0.139	=
	GOA	7	3	1.478	0.139	=

**Table 8:** Comparison of DAO and other algorithms for CBD problem.

Optimum Attributes							
Algorithms	*z*_1_	*z*_2_	*z*_3_	*z*_4_	*z*_5_	Optimum Weight	CPU Runtime (s)
DAO	6.0112	5.1211	4.8221	3.2114	2.1510	**1.3302**	1.986
COA [48]	6.0172	5.3071	4.4912	3.5081	2.1499	1.3999	2.001
AO [9]	5.8492	5.5413	4.3778	3.5978	2.1026	1.3596	1.926
ROA [49]	6.0156	5.1001	4.303	3.7365	2.3183	1.3456	1.256
GWO [50]	5.9956	5.4121	4.5986	3.5689	2.3548	1.3586	1.112
WOA [6]	5.8393	5.1582	4.9917	3.693	2.2275	1.3467	**0.606**
SCA [51]	5.9264	5.9285	4.5223	3.3267	1.9923	1.3581	1.111

Note: bold is used to indicate better result.

**Table 9:** Comparison of DAO and other algorithms for WBD problem.

Optimum Attributes						
Algorithms	H	L	T_T_	B_B_	Optimum Cost	CPU Runtime (s)
DAO	0.2138	3.2154	9.0275	0.2052	1.6960	2.410
COA [48]	0.2456	3.2563	9.0403	0.2057	1.6963	2.031
AO [9]	0.1631	3.3652	9.0202	0.2067	**1.6566**	2.399
SSA [44]	0.2057	3.4714	9.0366	0.2057	1.7249	2.121
WOA [6]	0.2054	3.4843	9.0374	0.2062	1.7305	**1.037**

Note: bold is used to indicate better result.

**Table 10:** Comparison of DAO and other algorithms for PVD problem.

Optimum Attributes						
Algorithms	$T_{S}$	$T_{H}$	r	$L_{CS}$	Optimum Cost	CPU Runtime (s)
**DAO**	0.7885	0.3254	42.3275	189.892	5877.1000	2.432
**COA** [48]	0.7437	0.3705	40.3238	199.9414	5735.2488	2.356
**AO** [9]	1.0540	0.1828	59.6219	38.8050	5949.2258	2.222
**GWO** [50]	0.8125	0.4345	42.0891	176.7587	6051.5639	1.345
**ROA** [49]	0.7295	0.2226	40.4323	198.5537	**5311.9175**	2.252
**RSA** [5]	0.8071	0.4426	43.6335	142.5359	6213.8317	1.125
**WOA** [6]	0.8125	0.4375	42.0982	76.6389	6059.7410	**0.872**

Note: bold is used to indicate better result.

## Data Availability

Since no datasets were created or examined in the current investigation, data sharing is not relevant to this topic.
